# Supplementary material for: Compressional pathways of α-cristobalite, structure of cristobalite X-I, and towards the understanding of seifertite formation
Source: Nat Commun. 2017 Jun 7;8:15647. doi: 10.1038/ncomms15647 (PMC5467234; doi:10.1038/ncomms15647)
Supplement: Supplementary Information — Supplementary Figures, Supplementary Tables and Supplementary References [file ncomms15647-s1.pdf]

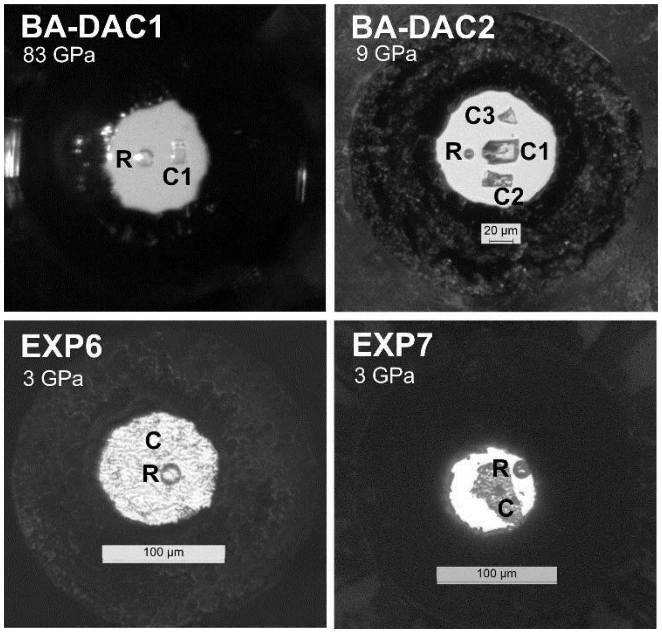

Supplementary Figure 1: Photographs of the representative DAC experiments. We carried out seven compression - decompression experiments on single crystals and powders of α-cristobalite up to variable pressures (max. ~83 GPa). All experiments were carried out at room temperature. R stands for ruby; C1, C2 and C3 are single crystals of cristobalite; C marks cristobalite powder. BA-DAC 1 & 2 are the single crystal set-ups analyzed at synchrotron. Same as these were experiments EXP1 and EXP4. EXP 6 & 7 are powder samples analyzed *in situ* using Raman spectroscopy, and upon quenching by in-house X-ray diffractometry and TEM. EXP 5 was DAC setup similar to that of BA-DAC1, whereby the experiment was carried out by a rapid pressure load to 9 and then 12 GPa.

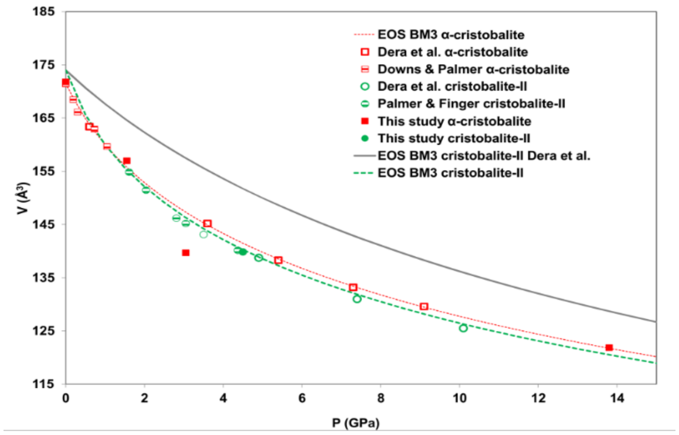

Supplementary Figure 2: Unit-cell volume compressibility. α-cristobalite (red) and cristobalite-II (green; half unit cell volume) are from this study, compared to various literature data[1,2,3]. Data with full symbols stem from this study.

Supplementary Table 1: Unit cell parameters and refinement statistics of α-cristobalite and cristobalite-II.

	P_{ruby} [GPa]	a [Å]	b [Å]	c [Å]	β [°]	V [Å^3]	ρ [g/cm^3]	R_{int}	R_1
**α-cristobalite ($P4_12_12$)**									
in air[#Bruker]	0.00	4.9806(15)		6.9260(30)		171.82(11)	2.33	0.053	0.042
BA-DAC1[#ID09A]	1.55	4.8520(7)		6.6683(13)		156.99(7)	2.54	0.045	0.078
BA-DAC2[#ID27]	13.8	4.5202(15)		5.9645(16)		121.87(7)	3.27	0.021	0.067
**cristobalite-II ($P2_1/c$)**									
BA-DAC1[#ID09A]	4.50	8.0750(60)	4.5745(3)	8.9600(30)	121.03(7)	279.7(18)	2.88	0.046	0.150

Note: The structures were refined starting from the atomic coordinates of the respective polymorphs reported by Dera et el. (2011). Numbers in parentheses are the esd's in the last decimal place of each value.

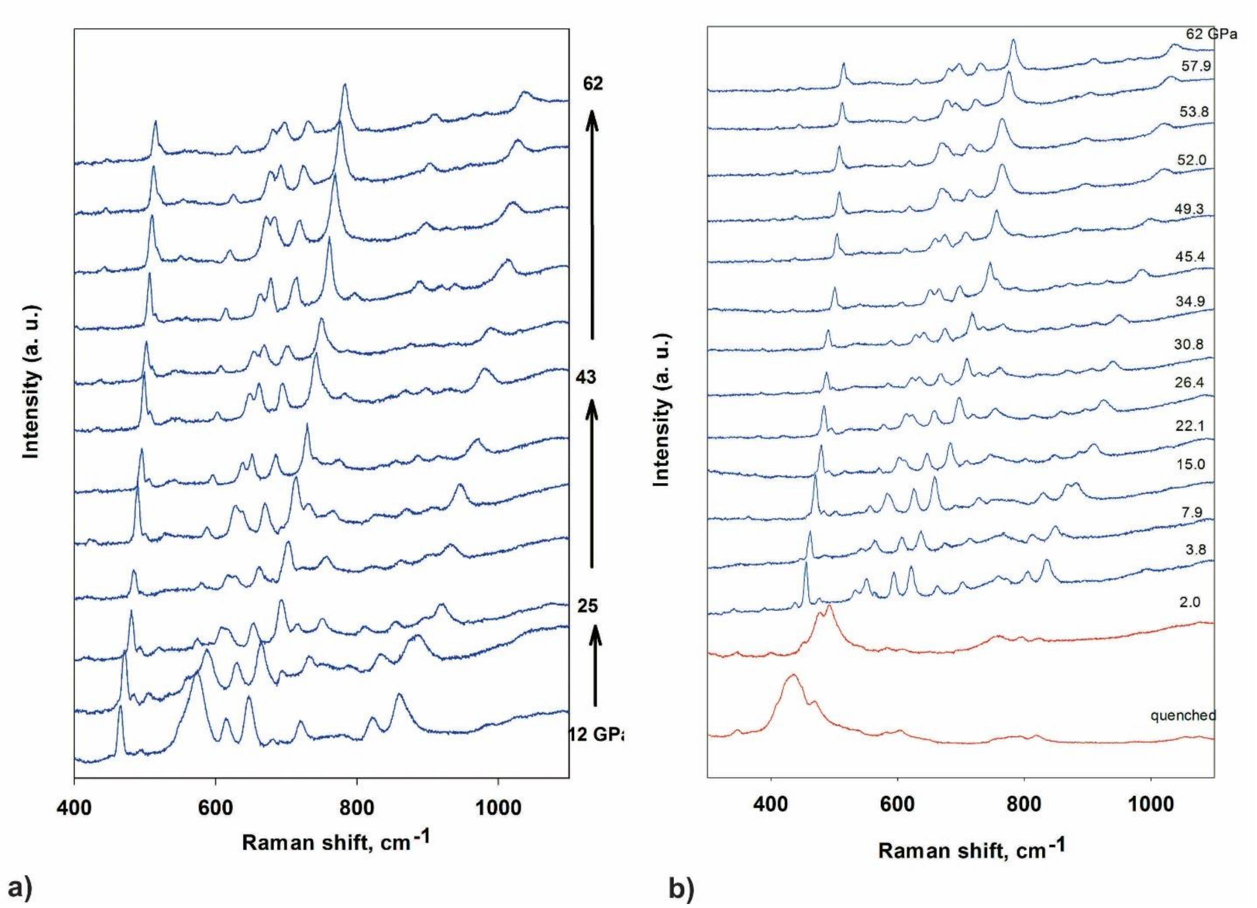

Supplementary Figure 3: ***In situ* Raman spectra of cristobalite X-I.** Spectra collected under compression (a) and decompression (b). The pressure (in GPa) is indicated on the right of the spectra. Upon decompression, cristobalite X-I transforms back to the starting α-cristobalite near 2 GPa. Due to similarity in spectra of α-cristobalite and cristobalite-II, we cannot be sure whether cristobalite-II is obscured on the decompression path starting from cristobalite X-I or it exists in a very narrow range. In either case, the hysteresis of ~8 GPa is suggestive of a first-order phase transition involved with the formation of the X-I phase. The pressure dependence of the Raman modes ($d\nu/dP$) of cristobalite X-I, where ν is the ambient-pressure phonon frequency and P is the pressure, ranges from 0.9(1)-3.2(1) cm^{-1} /GPa. These values match well with pressure-induced shifts reported for the only two octahedra-based SiO_2 forms studied by Raman spectroscopy, the rutile structured stishovite and its high-pressure, $CaCl_2$-structured polymorph. Based on the zero-pressure bulk modulus K_0=230(7) GPa determined in this study and using the formulation of the mode-Grüneisen parameter as $\gamma_\nu=(K_0/\nu)\ (d\nu/dP)$, we obtain an average of γ=0.7±0.1.

Supplementary Table 2:
Crystal chemistry of octahedral SiO_2 polymorphs

	Cristobalite X-I at 14.1 GPa		
	**Si1**	**Si2**	**Si3**
Si1-O (Å)	1.7663	1.7529	1.7867
O-Si1-O (°)	108.000	106.508	104.852
V_{oct} (Å^3)	7.3046	7.098	7.4285
Octahedral angle variance	13.066	27.3147	55.158
Mean octahedral quadratic elongation	1.0042	1.0082	1.0179

	Stishovite et 13.1 GPa[4]	Seifertite amb.[5]
	**Si1**	**Si1**
Si1-O (Å)	1.7561	1.7909
O-Si1-O (°)	108.000	106.088
V_{oct} (Å^3)	7.1338	7.5723
Octahedral angle variance	28.5395	26.711
Mean octahedral quadratic elongation	1.0083	1.0083

Supplementary Table 3: Experimental unit-cell parameters of cristobalite X-I at various pressures.

P_{ruby} [GPa]	*a* [Å]	*b* [Å]	*c* [Å]	*β* [°]	*V* [Å^3]	R_{int}
10.75(20)	6.664(11)	4.1066(7)	6.894(1)	98.35(5)	186.7(3)	0.05
14.10(10)	6.611(11)	4.0700(14)	6.853(5)	98.40(1)	182.4(3)	0.05
17.91(12)	6.582(1)	4.0443(6)	6.852(9)	98.13(4)	180.6(2)	0.08
22.95(34)	6.580(1)	4.0120(6)	6.802(13)	98.25(5)	177.7(3)	0.06
32.06(53)	6.556(15)	3.9654(8)	6.771(2)	98.16(8)	174.1(4)	-
47.94(10)	6.446(9)	3.8848(5)	6.670(1)	98.01(4)	165.4(2)	0.03
60.95(77)	6.393(1)	3.8371(7)	6.630(11)	98.05(5)	161.1(3)	0.07
69.65(55)	6.400(3)	3.8236(15)	6.527(4)	97.01(2)	158.5(7)	0.04

Note: Numbers in parentheses are the esd's in the last decimal place of each value.

Supplementary Table 4: Unit-cell parameters of cristobalite X-I obtained by *ab initio* calculations.

P [GPa]	*a* [Å]	*b* [Å]	*c* [Å]	*β* [°]	*V* [Bohr^3]	*V* [Å^3]
0.0	6.6415	4.1035	6.8863	98.0	1259.16	186.59
10.0	6.5808	4.0337	6.8240	98.0	1213.43	179.81
20.0	6.5289	3.9741	6.7684	98.0	1175.95	174.26
30.0	6.4832	3.9222	6.7179	98.0	1143.51	169.45
40.0	6.4419	3.8764	6.6719	98.0	1115.03	165.23
60.0	6.3694	3.7985	6.5898	98.0	1066.58	158.05
80.0	6.3066	3.7341	6.5179	98.0	1026.55	152.12
100.0	6.2509	3.6794	6.4535	98.0	992.47	147.07

Note: Numbers in parentheses are the esd's in the last decimal place of each value.

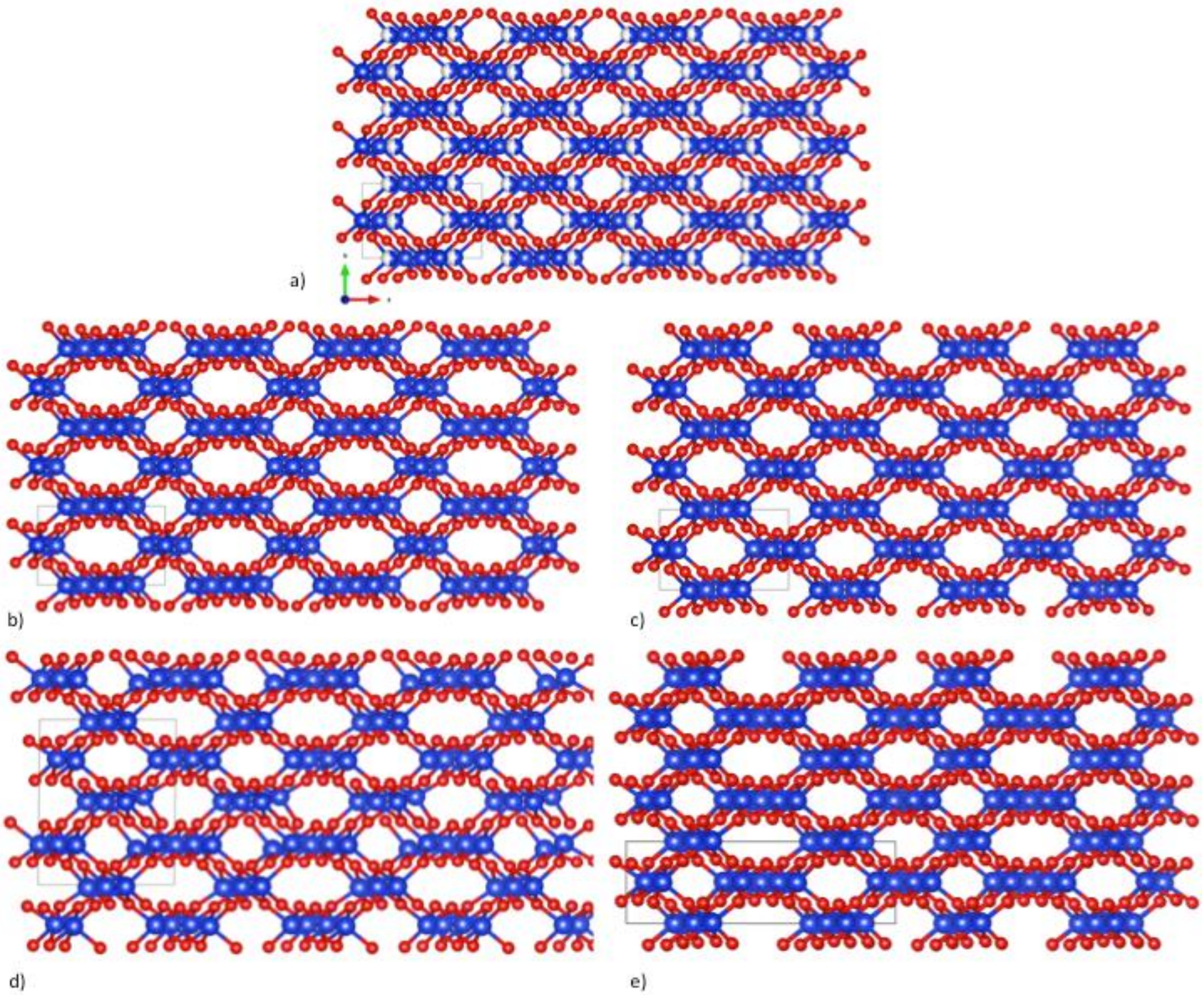

Supplementary Figure 4: Possible arrangements of the Si atoms in cristobalite X-I. The octahedra bind the columns parallel to the *c* axis. The sites that are equally probable to be occupied by Si are represented in (a) with the blue/white spheres. Two distinct local configurations (b) and (c) yield 5x3 and 4x4 octahedral columns, respectively, corresponding to the end-member ordered terms. Single-crystal X-ray refinements show the same probability for the two arrangements, while theoretical Raman calculations and comparison to measurements prefer 4x4 over 5x3. Further calculations were performed on two larger supercells alternating 4x4 and 5x3 arrangement along the y- (d) and x-axis (e).

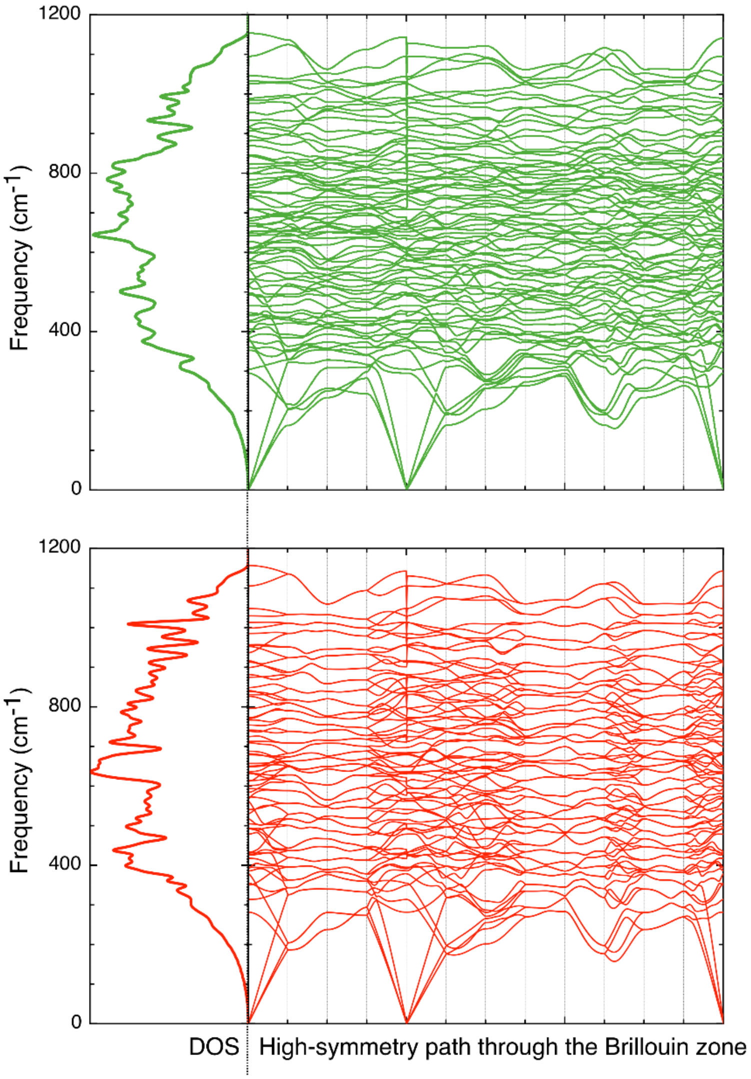

Supplementary Figure 5: Phonon dispersion curves (right panels) and their corresponding densities of states (left panels) of cristobalite X-I obtained at 40 GPa using the atomic arrangements shown in Supplementary Figure 5b (upper panels) and 5c (lower panels). The two phases are dynamically stable. Apart differences in degeneracy due to symmetry breaking, the dynamical properties are similar.

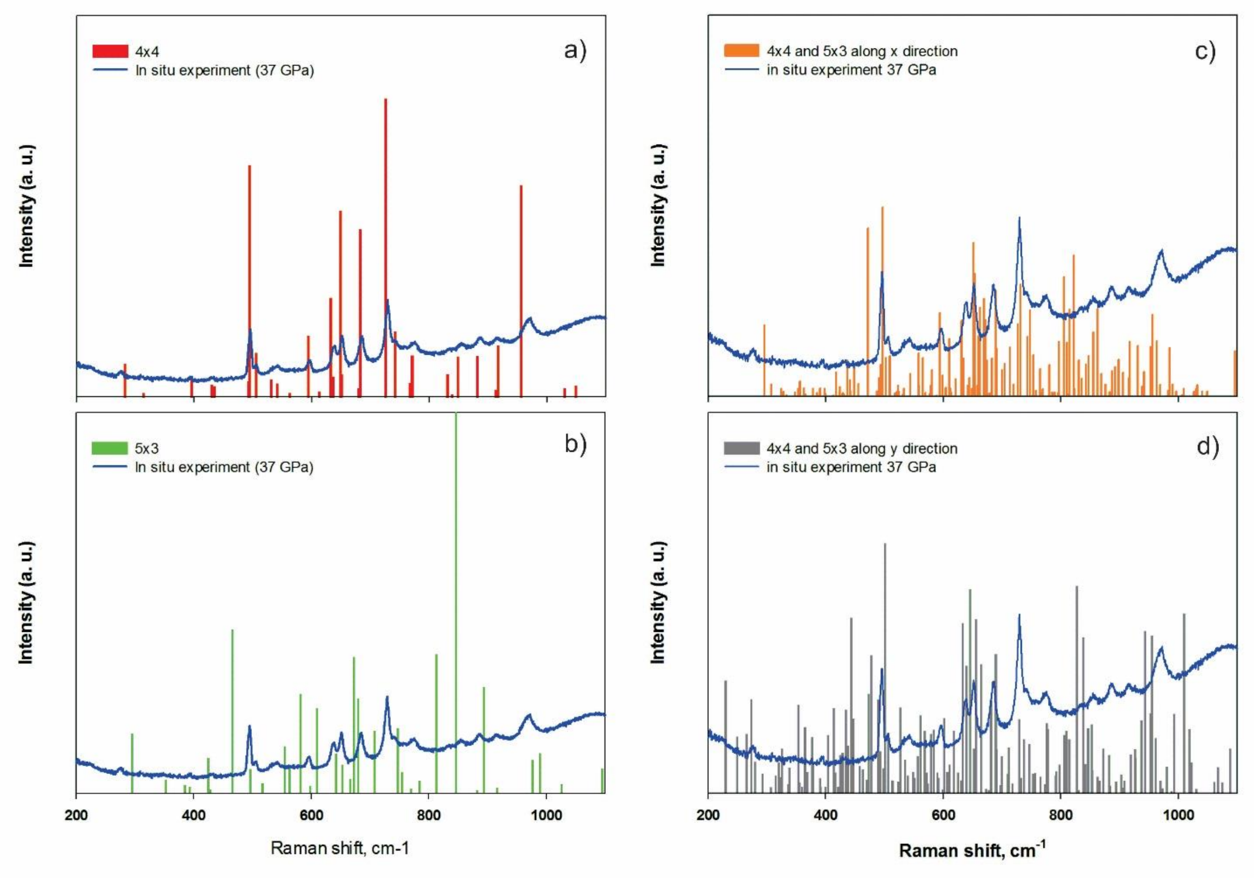

Supplementary Figure 6: Theoretical spectra for various cristobalite X-I configurations. Theoretical spectra are presented at 40 GPa and compared to the experimental spectra obtained at 37 GPa. The spectra of the two end-member arrangements, 5x3 and 4x4, shown in Supplementary Figures 4b and c, respectively, are plotted separately (panels a and b), as well as supercell structures alternating layers along x and y direction presented in Supplementary Figures 4e and d (panels c and d).

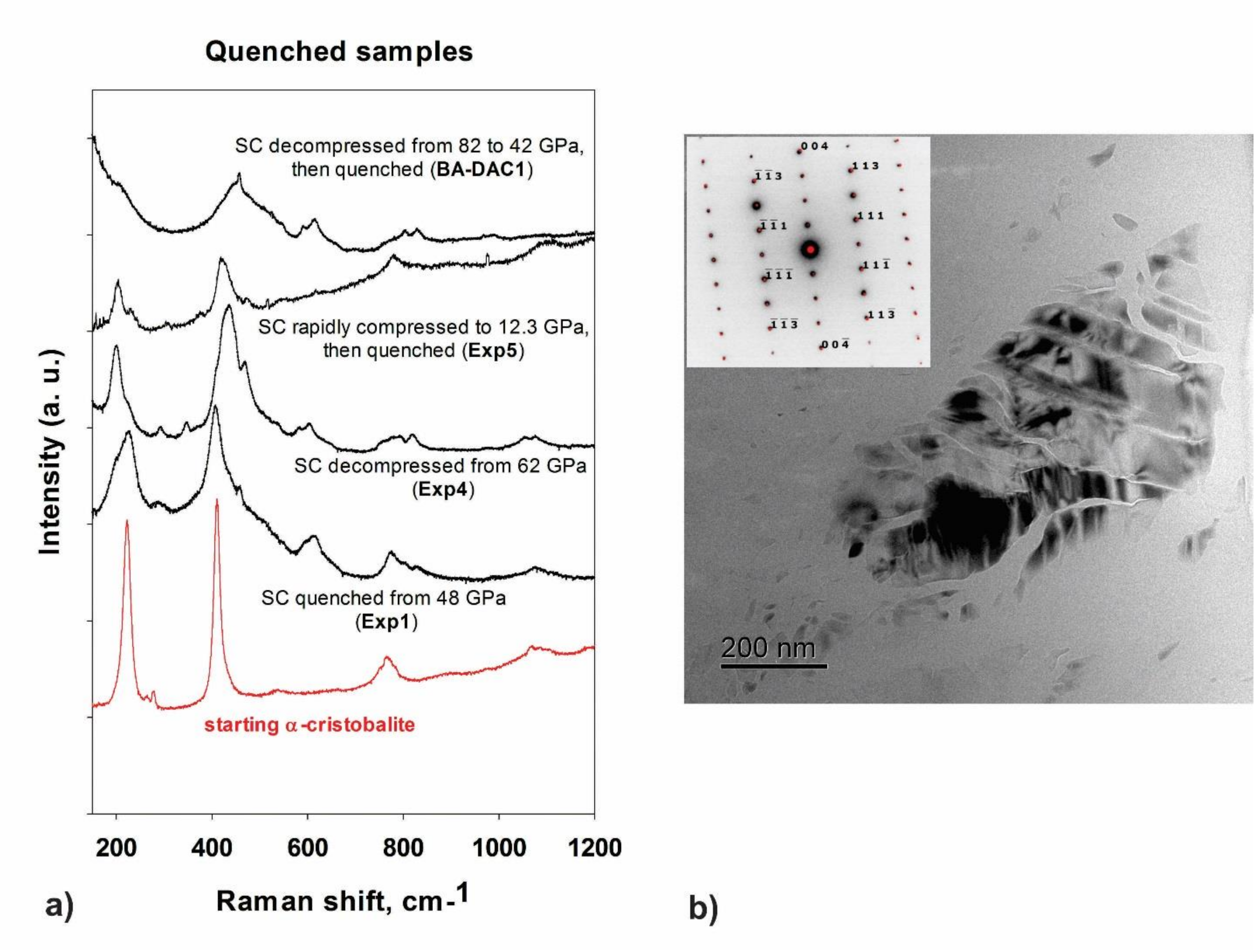

Supplementary Figure 7: Recovered single crystals. (a) Representative Raman spectra collected on the single crystals (SC) recovered from various pressures and under different compression rates, compared to the starting α-cristobalite. (b) The twinned crystalline grain is surrounded by an amorphous matrix and shows preferential amorphisation along the twin boundaries (dark grey to black). The inset shows a diffraction pattern indexed as α-cristobalite along the zone axis [-1 1 0], indicating that the entire initial grain had the same crystallographic orientation prior partial amorphization. Note that alltiny crystallites in the matrix are equally oriented. Black reflections in the inset are from this study, whereas the red reflections are simulated electron diffraction spots, starting from the structure reported by Dera et al[1]. Closed red symbols correspond to allowed reflections; open symbols are the forbidden reflections that appear in SAED due to dynamical diffraction effects.

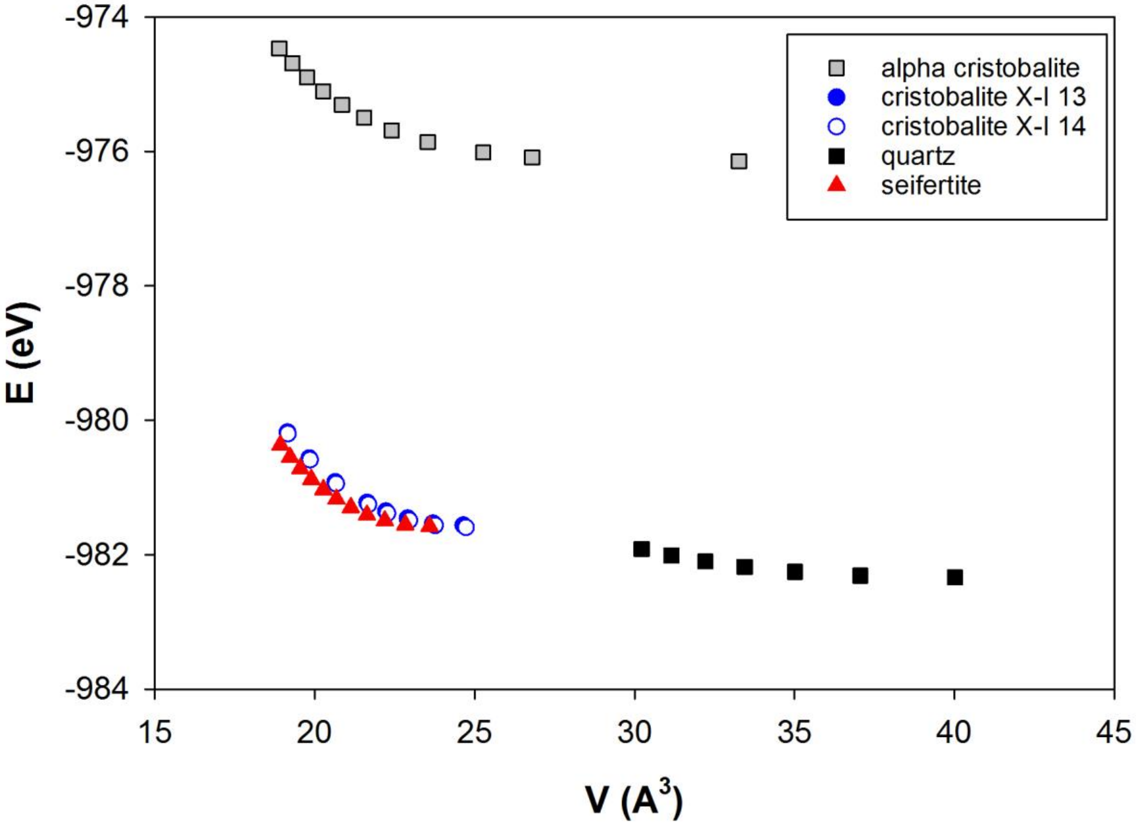

Supplementary Figure 8. Relative energy differences between selected tetrahedrally and octahedrally coordinated silica phases computed using Generalized-Gradient approximation (GGA) in the Perdew-Burke-Erzernhof formulation.

Supplementary Table 5: XRD and TEM reflections of quenched seifertite

Our study			Seifertite (El Goresy et al. 2008)		hkl (*Pbcn*)
XRD d [Å]	I/I_0	HRTEM d [Å]	d [Å]	I/I_0	
4.004	20				
3.558	40				
3.176	76		3.1807	70	110
2.96	13	2.94			
2.785	7				
2.395	4				
2.597	63	2.58	2.5963	67	111
		2.51	2.5231	11	020
2.249	21	2.29	2.2473	12	002
			2.2001	10	021
2.026	27		2.0485	2.5	200
1.954	100		1.9703	65	102
			1.9383	66	121
1.843	13		1.8354	13	112
			1.7485	2	211
1.677	7		1.6782	4	022
1.59	1		1.5903	4	220
1.557	68		1.556	33	130
1.515	58		1.5139	81	202
			1.4993	69	221
1.47	1		1.4704	1	131
1.439	1		1.3554	17	113
			1.2982	2	
1.27	70		1.2882	100	

Supplementary Table 6: Theoretical structure of cristobalite X-I at 40 GPa, 4x4 arrangement in P1 symmetry. Site occupancy is 1 for all atoms.

	x	y	z
Si1	0.489605	0.002793	0.50233
Si2	0.989605	0.497207	0.00233
Si3	0.357509	0.993021	0.11683
Si4	0.139104	0.502784	0.37525
Si5	0.639104	0.997216	0.87525
Si6	0.857509	0.506979	0.61683
Si7	0.271227	0.492983	0.76076
Si8	0.771227	0.007017	0.26076
O1	0.324849	0.219972	0.33275
O2	0.168545	0.722453	0.16769
O3	0.668545	0.777547	0.66769
O4	0.824849	0.280028	0.83275
O5	0.460165	0.222478	0.70989
O6	0.044162	0.698678	0.79833
O7	0.544162	0.801322	0.29833
O8	0.960165	0.277522	0.20989
O9	0.303872	0.719925	0.54483
O10	0.207392	0.252543	0.95941
O11	0.707392	0.247457	0.45941
O12	0.803872	0.780075	0.04483
O13	0.421328	0.752569	0.91818
O14	0.084543	0.301265	0.57924
O15	0.584543	0.198735	0.07925
O16	0.921328	0.747431	0.41818

Note: *The structure will be deposited on WURM database*

References cited in Supplementary Material:

1. Dera, P. et al. New insights into the high-pressure polymorphism of SiO2 cristobalite. *Phys. Chem. Miner.* **38,** 517–529 (2011).

2. Downs, R. & Palmer, D. The Pressure Behavior of Alpha-Cristobalite. *Am. Mineral.* **79,** 9–14 (1994).

3. Palmer, D. & Finger, L. Pressure-Induced Phase-Transition in Cristobalite - an X-Ray-Powder Diffraction Study to 4.4 Gpa. *Am. Mineral.* **79,** 1–8 (1994).

4. Ross, N. et al. High-Pressure Crystal-Chemistry of Stishovite. *Am. Miner.* **75,** 739–747 (1990).

5. Goresy, A. E. *et al.* Seifertite, a dense orthorhombic polymorph of silica from the Martian meteorites Shergotty and Zagami. *Eur J Mineral* **20,** 523–528 (2008).
